# Systematic Functional Comparative Analysis of Four Single-Stranded DNA-Binding Proteins and Their Affection on Viral RNA Metabolism

**DOI:** 10.1371/journal.pone.0055076

**Published:** 2013-01-24

**Authors:** Haiyan Shi, Yonghui Zhang, Guohui Zhang, Jinlei Guo, Xun Zhang, Haiyan Song, Jianxin Lv, Jimin Gao, Yuepeng Wang, Litian Chen, Yue Wang

**Affiliations:** 1 National Institute for Viral Disease Control and Prevention, China Center for Disease Control and Prevention, Beijing, China; 2 Zhejiang Provincial Key Lab for Technology and Application of Model Organisms Key Laboratory of Laboratory Medicine, Ministry of Education, Wenzhou Medical College, Wenzhou, China; 3 Department of Liver Transplantation Surgery, Xinhua Hospital, School of Medicine, Shanghai Jiaotong University, Shanghai, China; 4 Department of Endocrinology and Metabolism, The Second Affiliated Hospital of Harbin Medical University, Harbin, China; 5 Department of Cardiology, Xinhua Hospital, School of Medicine, Shanghai Jiaotong University, Shanghai, China; University of Hong Kong, Hong Kong

## Abstract

The accumulation of single-stranded DNA-binding (SSB) proteins is essential for organisms and has various applications. However, no study has simultaneously and systematically compared the characteristics of SSB proteins. In addition, SSB proteins may bind RNA and play an unknown biological role in RNA metabolism. Here, we expressed a novel species of SSB protein derived from *Thermococcus kodakarensis* KOD1 (KOD), as well as SSB proteins from *Thermus thermophilus* (TTH), *Escherichia coli*, and *Sulfolobus Solfataricus* P2 (SSOB), abbreviated kod, tth, bl21, and ssob, respectively. These SSB proteins could bind ssDNA and viral RNA. bl21 resisted heat treatment for more than 9 h, Ssob and kod could withstand 95°C for 10 h and retain its ssDNA- and RNA-binding ability. Four SSB proteins promoted the specificity of the DNA polymerase in PCR-based 5- and 9-kb genome fragment amplification. kod also increased the amplification of a 13-kb PCR product, and SSB protein–bound RNA resisted Benzonase digestion. The SSB proteins could also enter the host cell bound to RNA, which resulted in modulation of viral RNA metabolism, particularly ssob and bl21.

## Introduction

Nucleic acid replication, repair, recombination, transcription, and translation play a critical role in genome metabolism in organisms [1]. These processes are rigorously and orderly integrated at the molecular level [1]. SSB proteins play a key role in the integration of metabolic pathways by helping target relevant enzymes to the sites of on-going genome maintenance programs and ensuring that the enzymes complete their biological function effectively and accurately [2]–[5]. SSB proteins are typically tetrameric or dimeric, and each polypeptide is composed of an N-terminal DNA-binding domain, which presents a narrow ssDNA-binding cleft studded with residues that make numerous interactions with a small number of nucleotides in a relatively compact substructure [6]. They also contain a glycine and proline-rich region, as well as a flexible C-terminal tail essential for protein interactions [6].

SSB proteins are present in all three branches of organisms and in viruses [2]. Recently, several studies on the biological function and application of SSB proteins have been performed. SSB proteins protect the vulnerable single-stranded DNA (ssDNA) from nucleolytic digestion and prevent hairpin formation, thus keeping ssDNA in a suitable conformation for the action of enzymes involved in processes such as DNA replication, repair, recombination, and transcription [7]. Furthermore, SSB proteins are involved in physical interactions with several proteins that play important roles in those pathways, and thus are critical for essential life activities [8]. SSB proteins are known to increase the efficiency and fidelity of polymerase chain reaction (PCR) [9], and also to prevent the formation of primer dimers [10]. The SSB protein of TTH also interacts efficiently with RNA, resulting in a dramatic increase in the size of the cDNA synthesised by the reverse transcriptase activity of TTH DNA polymerase [9]. The SSB protein of *Escherichia coli* has the following functions; targeting restriction endonuclease digestion to any restriction enzyme site in cloned ssDNA via DNA oligo splints [11], improving the DNA sequencing results through regions with strong secondary structure [12], and enhancing RNA transcription [13]. The unique ability of SSB proteins to bind ssDNA but not double-stranded DNA (dsDNA) allows for the efficient separation of the three types of DNA molecules in the PCR reaction mixture, which makes it an indispensable tool in the development of nucleotide aptamers [2]. The affinity of SSB towards ssDNA has been successfully utilised to detect hybridisation on gold surfaces by surface plasmon-resonance imaging [14].

Although numerous applications of SSB proteins have been reported, a systematic functional comparative analysis of SSB proteins derived from different species has not yet been performed. Thus, it remains possible that SSB proteins bind single-stranded RNA. The SSB proteins may also have novel applications based on other biological characteristics. Also, differences between the SSB proteins derived from thermophilic bacteria and *E. coli* remain unknown. In this study, we compared the biological functions of SSB proteins derived from the thermophilic bacteria TTH, SSOB, and KOD, as well as *E. coli* BL21 (DE3). Overall, our results increase our understanding of the SSB proteins.

## Materials and Methods

### SSB Protein Expression and Purification

We selected three SSB proteins derived from the thermophilic bacteria *Thermus thermophilus* HB8 (AP008226.1), *Sulfolobus Solfataricus* P2 (AE006641.1), and *Thermococcus kodakarensis* KOD1 (AP006878.1), and one SSB protein from *Escherichia coli* BL21 (AM946981) termed tth, ssob, kod and bl21, respectively. To express these four SSB proteins, the coding sequences of tth, ssob and kod were synthesised by Biovisualab Ltd (Biovisualab, Shanghai, China) with a His-tag at their N-termini, and were inserted into the pET11a expression vector (Merck, Whitehouse Station, NJ). The open reading frame encoding bl21 was cloned by PCR using *E. coli* BL21 (DE3) chromosomal DNA as template with primers (forward, 5′-TATACATATGGCTCACCACCACCACCACCACGCCAGCAG AGGCGTAAACAAG-3′, reverse, 5′-TGGTGGTGCTCGAGTCATC AGAACGGAATGTCATCATC-3′). A high-fidelity DNA polymerase (HiFiFast) (Biovisualab, Shanghai, China) was used. The PCR product with a His-tag was inserted into a pET21a expression vector (Merck, Whitehouse Station, NJ) after digestion with *Nde*I and *Xho*I (New England Biolabs, Ipswich, MA). After sequence confirmation, the correct plasmids were used for SSB protein expression. Based on the supporting rare codon, tth was expressed in BL21 (DE3) competent cells and the remaining kod, ssob, and bl21 were expressed in Transetta (DE3) chemically competent cells (TransGen Biotech, Beijing, China).

Cells were grown at 37°C in LB medium containing ampicillin (80 mg/L) or chloramphenicol (34 mg/L) to an OD_600_ of 0.8. IPTG was then added to a final concentration of 1 mM. The cells were harvested after 4 h induction by centrifugation at 3,000 rpm for 30 min in a Hitachi centrifuge (Hitachi, Japan), and the pellet was resuspended in 50 mL of buffer (20 mM Tris–HCl pH 8.0, 0.5 M NaCl, 1 mM PMSF and 0.02% Triton-X100), and then disrupted by sonication. Insoluble debris was removed by centrifugation at 12,000 rpm for 20 min in a Hitachi centrifuge (Hitachi, Japan). The supernatant was heat-treated at 70°C for 30 min and denatured mesophilic proteins were discarded by centrifugation at 12,000 rpm for 20 min in a Hitachi centrifuge (Hitachi, Japan). The clarified supernatant was loaded onto a Ni^2+^ Sepharose column (GE Healthcare, Waukesha, WI), and bound proteins were washed and eluted with 5–120 mM imidazole. The eluted fractions containing protein were detected by sodium dodecyl sulphate polyacrylamide gel electrophoresis (SDS-PAGE). Target proteins were collected and dialysed against 40 mM Tris–HCl (pH 8.0) and 200 mM NaCl. The concentration of purified protein was determined using a spectrophotometer (Eppendorf, Hamburg, Germany) with the absorption coefficient at 280 nm. A series of quantitative albumin was used as a reference. After filtration through a 0.45-mm Durapore polyvinylidene fluoride (PVDF) membrane filter (Millipore Ireland, Cork, Ireland), 50% (v/v) glycerol was added to each of the SSB proteins and then stored at −20°C for further use.

### SDS-PAGE and Native-PAGE Analysis

To confirm the protein molecular weight and purity, 5 µg of each SSB protein was applied to 12.5% running gel containing 0.1% SDS under reducing conditions for SDS-PAGE analysis. To verify the polymerisation form of these SSB proteins, 5 µg of each SSB protein was loaded onto 3.5–5–12% non-denaturing gradient polyacrylamide gels for electrophoresis with a NativeMarker™ protein marker (Life Technoloies, Carisbad, CA). ssob is a strongly alkaline protein, it’s isoelectric point = 8.464, with positive charge at PH 8.3 buffer and will move to anode. To visualize the ssob in a native-PAGE, we lowered the pH of the running buffer and gel to 7.4 and reversed the negative and positive pole. All gels were stained with Coomassie brilliant blue R-250 (Tsingke, Beijing, China).

### ssDNA and Viral RNA Binding Analysis

To verify the ssDNA binding activity of the four SSB proteins, a 146- or 537-bp PCR product was denatured at 100°C for 10 min and cooled on ice rapidly to obtain ssDNA. ssDNA (250 ng) was incubated with 1–5 µg of each of the SSB proteins in 100 mM Tris-HCl (pH 8.0) and 0.2 mM EDTA binding buffer at room temperature for 5 min. Then, the protein ssDNA mix was analysed by native-PAGE analysis. To visualise the ssDNA–SSB protein complex, we first performed AgNO_3_ staining since this method stains both DNA and protein. The gels were fixed with 10% acetic acid for 20 min, rinsed three times with water for 2 min, impregnated with AgNO_3_ (1 g/L) and HCOH (1.5 mL/L) for 30 min, rinsed again with water briefly, and visualised using developing solution (30 g/L Na_2_CO_3_, 1.5 mL/L HCOH, and 2 mg/L Na_2_S_2_O_3_^.^5H_2_O) for 2–10 min. The ssDNA was clearly stained red [15]. Although this method can be used to identify DNA and proteins, it cannot efficiently identify the ssDNA–protein complex. To complement this method, we stained the gel with Coomassie brilliant blue R-250 since the protein ssDNA complex migrated slower than protein alone. The protein binding activity could be analysed using this method, and relative dsDNA was used as a specific control.

For RNA binding analysis, 120 ng of *in vitro* transcribed hepatitis C virus (HCV) RNA was incubated with 1.5 µg each of the four SSB proteins in 100 mM Tris-HCl (pH 8.0) and 0.2 mM EDTA binding buffer at room temperature for 5 min. The denatured HCV RNA was obtained by heating the RNA at 65°C for 5 min. RNA–SSB protein complexes were applied to a 1.2% TAE agarose gel. After electrophoresis, RNA or RNA protein complexes were stained with ethidium bromide.

To determine their heat resistance, the SSB proteins were incubated in water at 95°C for 5, 120, 240, or 600 min. Their binding activity was re-confirmed, as described above.

### Benzonase Challenge Experiments

To determine if the SSB proteins could protect the RNA against nuclease attack *in vitro*, we examined the threshold dose of Benzonase (Novagen, Germany) with 120 ng of HCV RNA, according to the manufacturer’s protocol. A total of 3.2×10^−3 ^U of Benzonase could digest 120 ng of HCV RNA at room temperature within 30 min, while 3.2×10^−2 ^U could completely digest the HCV RNA. To challenge the protection effect, 1.5 µg of each of the SSB proteins were added to bind RNA or denatured RNA. Next, 3.2×10^−3 ^U and 3.2×10^−2 ^U of Benzonase were added and incubated at room temperature for 30 min. The protection effect was visualised by 1.2%TAE agarose gel electrophoresis.

### PCR Specificity Analysis

To evaluate the effect of SSB proteins on PCR specificity, the human beta globin gene was used as template to amplify a 5-kb (Forward primer: 5′-GGCCAGC AGCTGCTGTGCTCAATTCCTCGC-3′, reverse primer: 5′- GTGTGCAGTTCAAGGCCCTGTAGTTGCT G-3′), 9 kb (forward primer: 5′-GGCCAGCAGCTGCTGTGCTCAATTCCTCGC-3′, reverse primer: 5′- CCTCTCCTCTCTTACTCATCCCATCACGTATGCC-3′) and a 13-kb (forward primer: 5′-GGCCAGCAGCTGCTGTGCTCAATTCCTCGC-3′, reverse primer: 5′-CTGGC CAGAACTGCTCATGCTTGGACTATGG-3′) PCR fragment. The HiFiFast DNA polymerase (Biovisualab, Shanghai, China) without any modifications was selected to verify the effect of SSB proteins on PCR specificity. The PCR reaction conditions were optimised as follows; 100 ng of template DNA, 200 nM each of the dNTPs, 200 nM each of the primers, 2.5 µM MgCl_2_, and 2.5 U of HiFiFast DNA polymerase (final concentration in 50 µL PCR reaction mix). The PCR cycling conditions were 2 min initial denaturation at 98°C, followed by 35 cycles of 20 s at 98°C, 3 min at 68°C, and 5 min final elongation at 72°C. To evaluate the SSB protein effect on PCR specificity, 0.5–2 ng of ssob, bl21, kod or tth was added to the PCR mix. The effect of SSB proteins was confirmed by 1% TBE agarose gel electrophoresis.

### In vitro Viral RNA Transcription and Transfection

A JFH1 HCV cDNA encoding plasmid (a gift from Dr. Wakita, Tokyo, Japan) was denatured with XbaI, and the 5′overhangs were removed by treatment with mung bean nuclease. Templates were then purified by digestion with proteinase K, two rounds of phenol-chloroform extraction, and ethanol precipitation, and then resuspended at 1 µg/µL. RNA transcription reactions contained 20 mM Tris-HCl (2-amino-2-(hydroxymethyl)-1,3-propanediol), pH 7.5; 5 mM sodium chloride, 9 mM magnesium chloride, 3 mM each nucleoside triphosphate, 20 units T7 RNA polymerase (Promega, WI), 12 units SUPERasin (Ambion, Austin, TX), and 0.05 µg/µL linear template. Following a 30 min synthesis at 37°C, RNAs were purified and DNase-treated with RNeasy minicolumns (Qiagen, Valencia, CA) and eluted in 2 mM sodium citrate, pH 6.4. RNA concentrations were measured using spectrophotometry and adjusted to 100 ng/µL. Aliquots (10 µL) of RNA were stored frozen at −80°C until use.

Huh7 cells (bought from RIKEN cell bank, Tsukuba, Ibaraki, Japan) were maintained in complete growth medium (Dulbecco’s modified Eagle medium (DMEM; Invitrogen) containing 10% heat-inactivated foetal calf serum (Invitrogen) and 0.1 mM non-essential amino acids. For RNA transfection, Huh7 cells were plated in 1.0×10^7^ cells/well 24 h prior to transfection and were ∼80% confluent at the time of transfection. DMRIE-C (4 µL) transfection reagent (Invitrogen) was added to 0.5-mL DMEM in a sterile polystyrene tube and mixed gently. Viral RNA (500 ng–1 µg) was then added, mixed and immediately added to the cells washed twice by DMEM prior to the transfection. The cells were incubated for 6 h at 37°C and in 5% CO_2_, and then the medium containing the transfection complexes was removed and replaced with complete growth medium (DMEM with 10% serum) for a further 48–72 h. To verify the effects of SSB proteins on viral RNA expression, 10 or 20 ng of each SSB protein was added to the transfection mix. To reduce systemic and technical errors, all transfections were performed in parallel.

### Immunocellularchemistry and Fluorescence-activated Cell Sorting (FACS) Assay

To confirm the effects of SSB proteins on viral RNA expression, the cells were harvested with 0.25% Trypsin-EDTA (Invitrogen) 48 h after the transfection. The cells were washed twice with PBS and subsequently fixed with 3.7% formaldehyde in PBS for 30 min at room temperature. After washing the cells again with PBS, the cells were blocked with PBS containing 2% normal serum for 1 h. The cells were then incubated with HCV-infected human serum (1∶50) (Written informed consent was obtained according to the guidance of the National Ethics Regulation Committee and approved by review board of China Center for Disease Control and Prevention. The patient agreed the usage of the sampled serum as primary antibody in this research) for 2 h at room temperature. Cells were washed three times with PBS and then incubated with FITC-labelled goat anti-human IgG (Molecular Probes, Eugene, OR) for 1 h at room temperature. After washing the cells twice with ice-cold PBS, the cells were submitted for FACS (BD Biosciences, San Diego, CA) to determine the number of HCV protein–positive cells.

To determine if the SSB proteins could co-enter the Huh7 cells together with the transfection complex, we performed immunocellularchemistry analysis in parallel with the FACS analysis. After transfection (48 h), the cells were washed twice with PBS and subsequently fixed with 3.7% formaldehyde in PBS for 30 min at room temperature. After washing the cells again with PBS, the cells were blocked with PBS containing 2% normal serum for 1 h. The cells were then incubated with a mouse anti-His antibody (Invitrogen) for 2 h at room temperature. Cells were washed three times with PBS and then incubated with FITC-labelled goat anti-mouse IgG (Molecular Probes, Eugene, OR) for 1 h at room temperature. After glass coverslips were coated with fluorescent mounting medium (Dako), SSB proteins in the host cell were examined using a confocal fluorescent microscope (Nikon, Japan).

### Statistical Analysis

The results are expressed as mean±standard deviation (SD) from at least 3 independent experiments. The statistical analysis was performed using Student *t* test or analysis of variance with *post ho*c Scheffe testing when appropriate. *p*<0.05 was considered statistically significant.

## Results

### Primary Sequence Analysis of Four SSB Proteins

**Figure 1:**
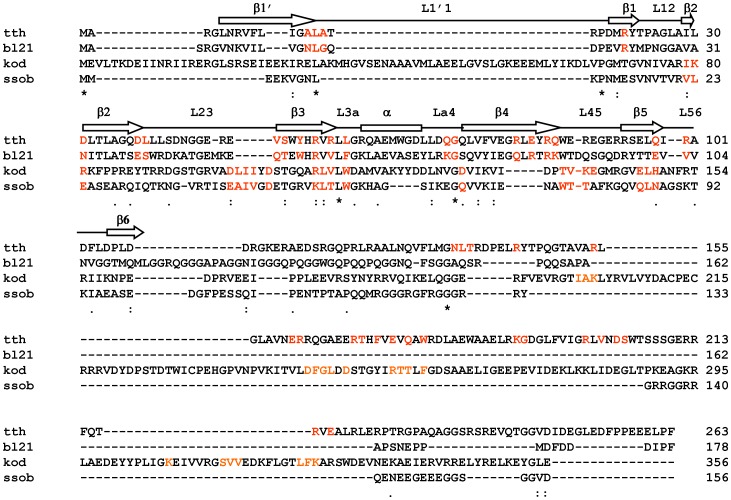
Primary sequence analysis of four SSB proteins. The amino-acid sequences of TTH, BL21, KOD and SSOB SSB proteins (NCBI access number: AP008226, AM946981, GM017008, and NP_343725, respectively) were aligned using a multiple alignment program for amino acid or nucleotide sequences (http://mafft.cbrc.jp/alignment/server/). The structure of the DNA-binding domain was marked according to reference 3. The conserved DNA-binding sites (amino acids labelled in red) were identified by the NCBI CD-Search & Batch CD-Search online programs. One dot represents a semi-conserved amino acid change, two dots represent a conserved amino acid change, and stars represent identical amino acids. All SSB proteins were abbreviated by the nomenclature of their ancestor bacteria.

To perform a systematic functional comparative analysis of four SSB proteins, the primary structure of TTH, BL21, KOD and SSOB SSB proteins (abbreviated as tth, bl21, kod, and ssob) were aligned using a multiple alignment program for amino acid or nucleotide sequences (http://mafft.cbrc.jp/alignment/server/). bl21 contained a ssDNA binding domain, a glycine and proline-rich region, and a C-terminal tail, as reported previously (Figure 1). Although they were all derived from bacteria possessing similar single-strand DNA-binding function, the amino acid sequence alignments revealed significant differences. The complete amino acid sequences of tth, bl21, kod, and ssob were 263, 178, 356, and 156 residues in length, respectively (Figure 1 and Table 1). tth, kod, and ssob had lost a glycine and proline-rich region, and the sequence of the C-terminal tail varied significantly (Figure 1). The conserved DNA binding sites (amino acids labelled in red) were identified by NCBI CD-Search & Batch CD-Search online program. Base on conserved DNA binding analysis, the four SSB proteins were divided into two groups. Although TTH is a thermophilic bacterium and BL21 is an *E. coli* strain, the conserved DNA biding domains of both tth and bl21 shared the basic feature in primary sequence with that of the chymotryptic fragment of *E. coli* SSB protein (NCBI LOCUS:1EYG_D) (Figure 1). Meanwhile, kod and ssob displayed genetic relationship with the SSB protein of a SSOB (NCBI LOCUS: 1O7I_B) (Figure 1). tth and kod contained an extra single-strand DNA-binding domain within their C-terminal region compared to bl21 and ssob (Figure 1).

**Table 1:** Summary of SSB proteins properties.

	ssob	tth	kod	bl21
Amino acid composing (aa)	156	263	356	178
Molecular weight of monomer (kDa)	20.0	29.8	41.6	19.8
Functional units	tetramer	dimer	dimer	tetramer
Binding to ssDNA	Yes	Yes	Yes	Yes
95°C heat-resistance	>10 h	4 min	>10 h	9 h
Enhance target DNA synthesis	Yes	Yes	Yes	Yes
Reduce PCR by-products	Yes	Yes	Yes	Yes
Binding to ssRNA	Yes	Yes	Yes	Yes
Protect RNA against nuclease digestion	Partially	Noneffectively	Effectively	Effectively
enhance viral RNA transduction and expression	Yes	Yes	Yes	Yes

Note: h, hour; min, minute.

### Expression and Purification of SSB Proteins

Four SSB proteins encoding genes with a 6× his-tag at the 5′ terminus were inserted into the pET expression system. Suitable competent cells were transformed with the expression plasmids, and robust and stable expression clones were selected to yield SSB proteins after IPTG induction. SSB proteins were purified by affinity chromatography after cell lysis at 70°C for 20 min, excluding bl21, which did not undergo the heating stage.

**Figure 2:**
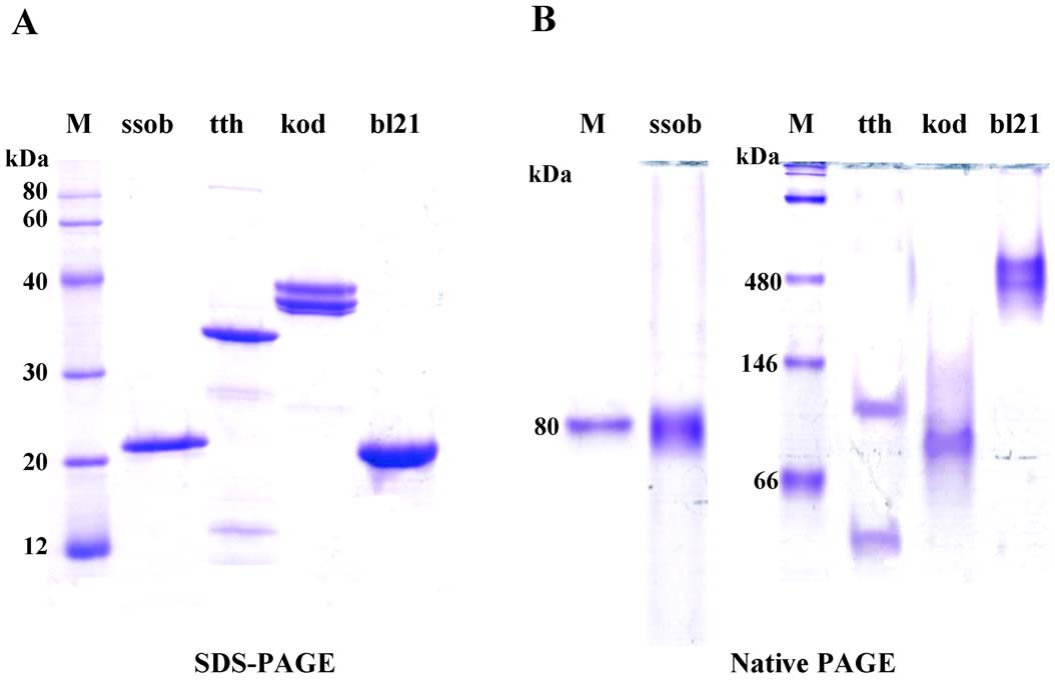
SDS-PAGE and native PAGE analysis of SSB proteins. A. Purity and protein molecular weight were visualised by SDS-PAGE in a 12.5% polyacrylamide gel containing 0.1% SDS under reducing conditions. B. Functional SSB protein polymers were notarised by 3.5–5–12% non-denaturing gel electrophoresis.

To visualise their purity and molecular weight, all SSB proteins were applied to SDS-PAGE analysis in a 12.5% polyacrylamide gel containing 0.1% SDS under reducing conditions. The molecular weights of ssob, tth, and bl21 were 20, 29.8, and 19.8 kDa, respectively (Table 1), which is consistent with previous reports [9], [13], [16]. All three proteins showed single clear bands (Figure 2A). The predicted molecular weight of kod was approximately 41.6 kDa, and we found that KOD migrated at its expected molecular weight (near the 40 kDa marker). Notably, there were always two bands aggregated in the polyacrylamide gel, and this was not likely due to glycosylation since there were no N-X-S or N-X-T motifs in the kod primary sequence (Figures 1 and 2B). The functional units of ssob, tth, and bl21 are thought to be tetrameric, dimeric, and tetrameric, respectively [9], [13], [16]. Our native PAGE analysis was consistent with these reports, excluding bl21 which always migrated at approximately 480 kDa. Also, our data suggested that kod existed as a dimer (Figure 2, right, Table 1).

### Analysis of SSB Proteins Binding to ssDNA

**Figure 3:**
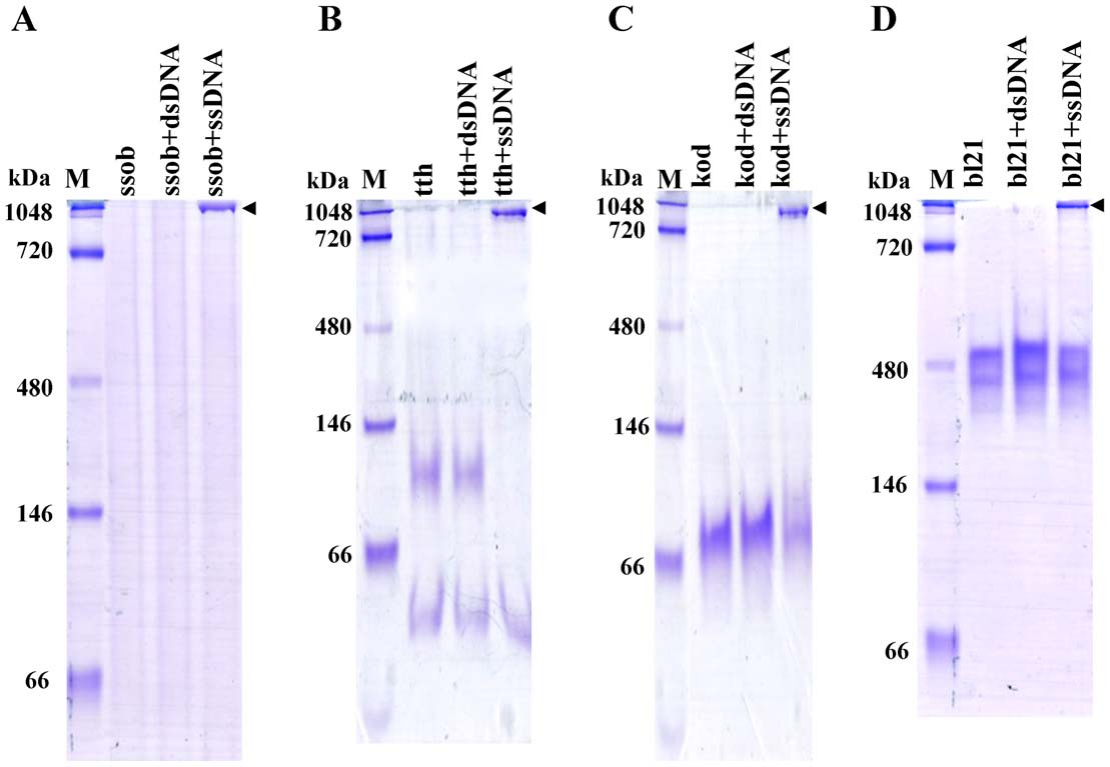
SSB proteins bind ssDNA. Non-denaturing gel electrophoresis (3.5–5–12%) was used to confirm SSB protein binding to ssDNA. Relative dsDNA was used as a binding control. M, monomer; D, dimer; T, tetramer. A, ssob binding ssDNA analysis. B, tth binding ssDNA analysis. C, kod binding ssDNA analysis. D, bl21 binding ssDNA analysis.

To confirm that SSB proteins bind DNA, PCR products (537 or 146 bp) were heated at 100°C for 10 min and cooled on ice rapidly to create ssDNA, Non-denaturing gel electrophoresis (3.5–5–12%) was used to confirm SSB protein-binding activity. The relative dsDNA was used as a binding specificity control. All SSB proteins displayed ssDNA binding characteristics. Due to ssob is a strongly alkaline protein, it’s isoelectric point = 8.464, with positive charge at pH 8.3 running buffer moving to anode, so we could not view the protein bands together with other SSBs. While, when ssob bound to the 537-bp ssDNA, the complex migrated around the 1048 kDa which suggested ssob could specifically bind to ssDNA and the isoelectric point was changed when ssob bound to ssDNA (Figure 3A). For tth alone or tth plus 537-bp dsDNA, the protein dimer migrated slower than 66 kDa while the monomer migrated faster than 66 kDa. When tth bound to the 537-bp ssDNA, the DNA protein complex also migrated at around 1048 kDa (Figure 3B). For kod alone or kod plus the 537-bp dsDNA, the protein dimer migrated slower than 66 kDa while the 537-bp ssDNA kod binding complex migrated faster than 1048 kDa, which suggested that this newly expressed SSB protein could bind ssDNA (Figure 3C). For bl21 alone or bl21 plus 537-bp dsDNA, the protein complex migrated close to 480 kDa. When the complex bound to the 537-bp ssDNA, the DNA protein complex migrated close to 1048 kDa (Figure 3D). When the 537-bp DNA fragment was replaced by a 146-bp DNA fragment, ssDNA binding activity was also observed (data not showed). Taken together, these data demonstrated that all four SSB proteins effectively and specifically bind ssDNA (Table 1).

### Heat-resistance Analysis of Four SSB Proteins

**Figure 4:**
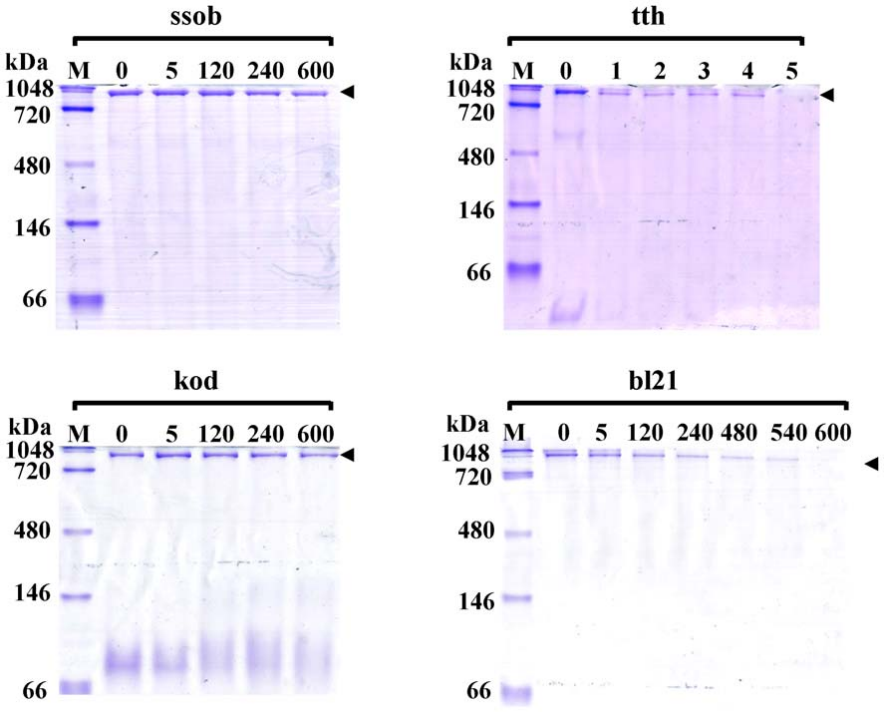
Heat-resistance analysis of four SSB proteins. Four SSB proteins were heated at 95°C for 1, 2, 3, 4, 5, 120, 240, and 600 min, respectively, and their binding activities were assessed by native PAGE. Black arrow indicates the SSB protein–ssDNA binding complex. Non-heat-treated SSB proteins were used as controls.

SSB proteins derived from thermophilic bacteria are thought to be heat resistant, as opposed to SSB proteins from *E. coli*. To confirm their heat-resistance, the four proteins were heated at 95°C for1, 2, 3, 4, 5, 120, 240, and 600 min, respectively, and their binding activity was assessed by native PAGE analysis. As reported by Perales *et al*., tth could not withstand heat treatment for more than 5 min [9] (Figure 4 and Table 1). When bl21 was heated for 4 h, it could still bind ssDNA, but it lost partial binding activity when further treated at 95°C for 5 h (Figure 4). Ssob could also withstand 95°C for 10 h (Figure 4 and Table 1). kod could resist heat treatment at 95°C for more than 10 h (Figure 4 and Table 1). Taken together, kod and ssob showed excellent heat-resistance, and SSB proteins derived from thermophilic bacteria were not always resistant to heat-treatment. Also, the SSB protein from *E. coli* could withstand heat treatment for more than 9 h.

### Effect of Four SSB Proteins on Long Fragment Human Genome Amplification

**Figure 5:**
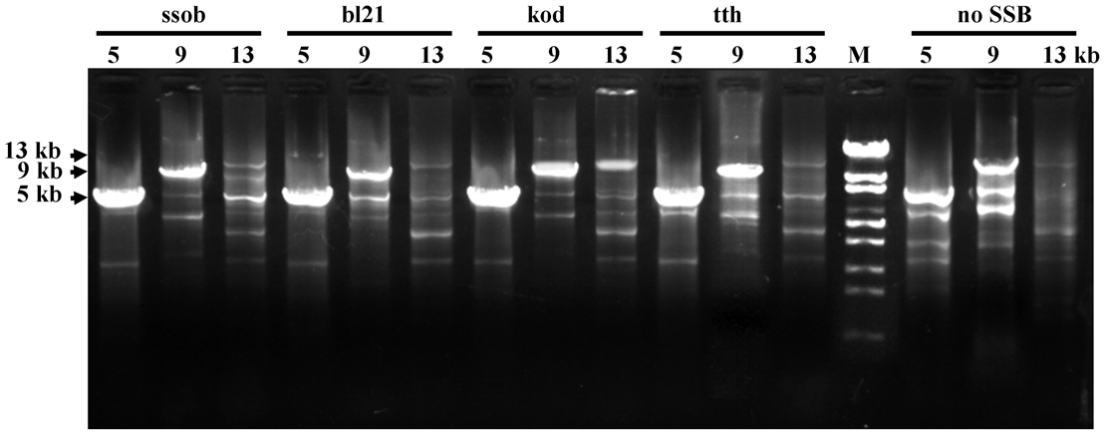
Effect of SSB proteins on long-fragment human genome amplification. Human beta globin gene fragments (5, 9, and 13 kb) were used as templates to assess the effect of SSB proteins on PCR amplification under optimised conditions.

SSB proteins are known to enhance target DNA synthesis and reduce PCR by-products [2]. To compare these abilities of the four SSB proteins, 5-, 9-, and 13-kb human beta globin gene fragments were used as templates for PCR. When PCR reactions did not include SSB proteins, although the DNA polymerase under optimised conditions could amplify the 5- and 9-kb fragments, the specific PCR product was contaminated with non-specific products (Figure 5 and Table 1). When SSB proteins were added, all four SSB proteins significantly enhanced amplification of the 5- and 9-kb target product and minimised the non-specific PCR products (Figure 5 and Table 1). Of the 13-kb human beta globin gene amplification, although ssob, bl21 and tth were slightly beneficial towards target product synthesis, kod most significantly enhanced target fragment amplification (Figure 5 and Table 1).

### Analysis of SSB Protein Binding to ssRNA

**Figure 6:**
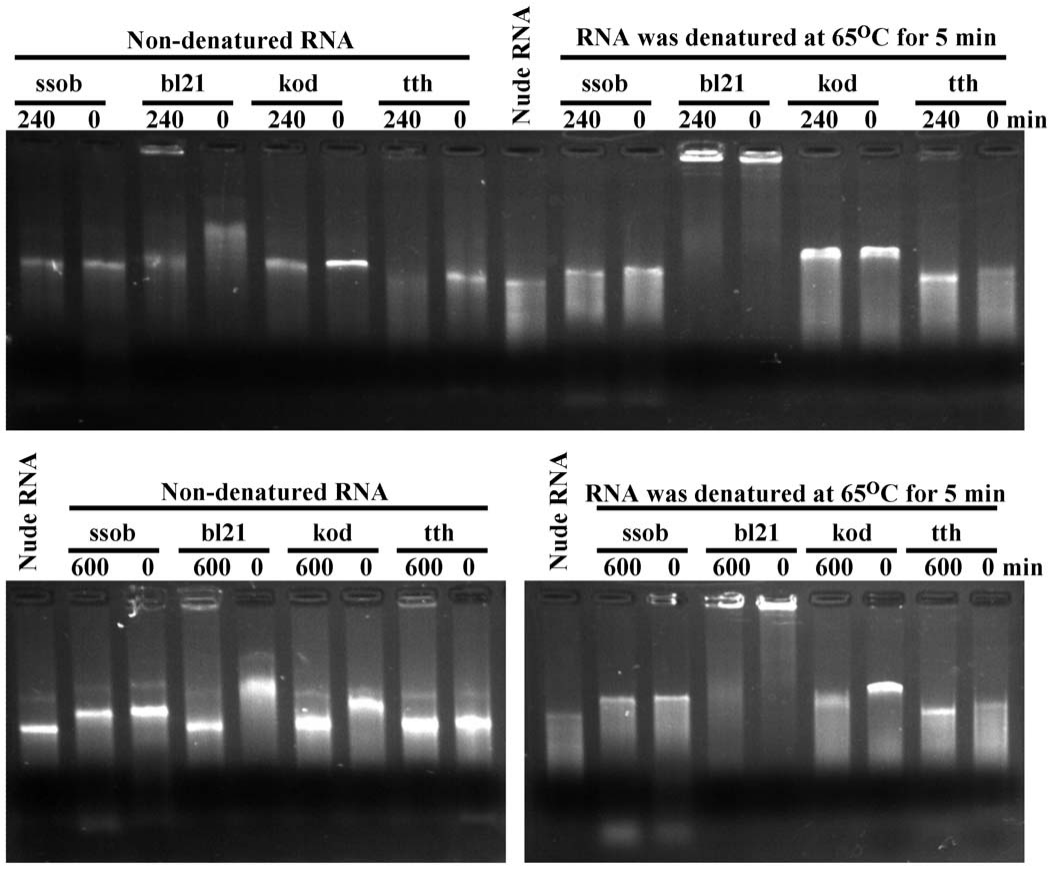
SSB proteins bind ssRNA. TAE agarose gel electrophoresis (1.2%) and an *in vitro*–transcribed HCV RNA were used to assess SSB protein-binding activity. Non-denatured RNA and denatured RNA were mixed with 240 and 600 min heat-treated SSB proteins. Zero minutes represents SSB proteins that were not heated, and nude RNA (denatured at 65°C for 5 min) represents RNA alone.

The specificity of SSB proteins to bind ssDNA but not RNA was thought to be determined by the specific structure of both SSB proteins and RNA [17]. To assess this specificity, an *in vitro* transcribed HCV RNA was used to interact with the four SSB proteins. The binding effect was visualised by 1.2% TAE agarose gel electrophoresis. Unexpectedly, when four SSB proteins associated with non-denatured RNA, the mobility was clearly slower than that of RNA alone (nude RNA), which suggested that SSB proteins bound ssRNA (Figure 6, upper left). When the four SSB proteins interacted with denatured RNA, bl21 and kod showed an increased binding efficiency (Table 1). Since numerous proteins bind to denatured HCV RNA, the mobility of the bl21 RNA complex was too heavy to migrate (Figure 6 upper right). To determine if heat treatment inhibited SSB protein-binding activity, all four SSB proteins were heated at 95°C for 240 and 600 min. ssob, kod, and bl21 showed unchanged binding characteristics, while tth lost its binding activity (Figure 6).

### SSB Proteins Protected RNA Against Nuclease Digestion

**Figure 7:**
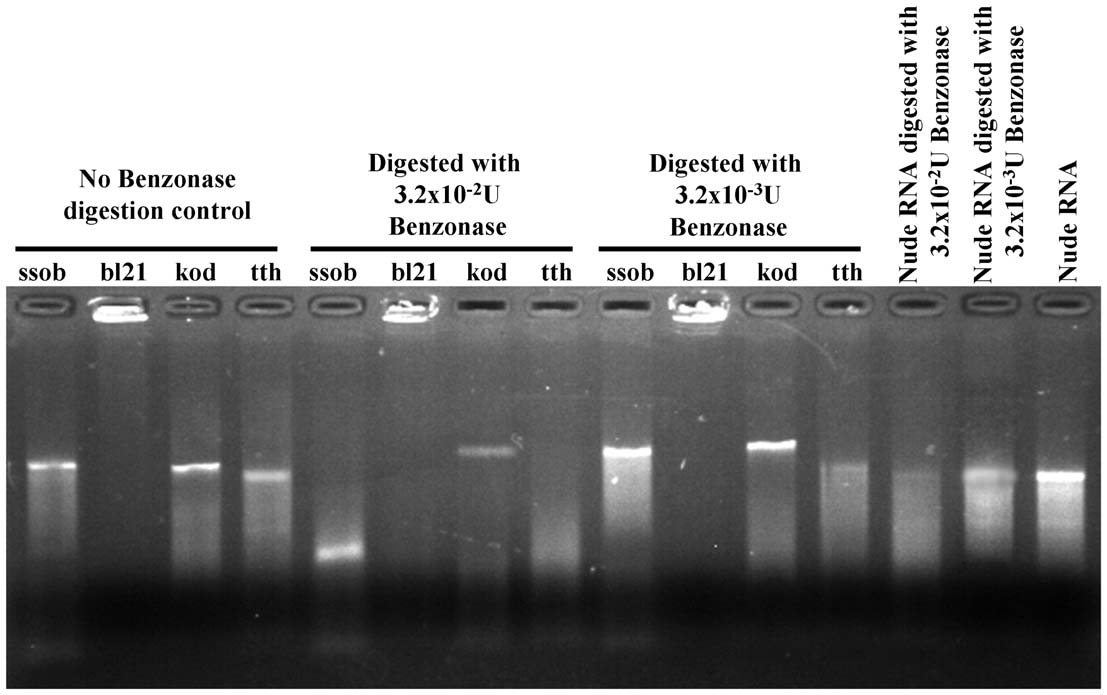
RNA protection assay. HCV RNA was denatured by incubation at 65°C for 5 min. The threshold and a 10-fold higher Benzonase doses were added to the SSB protein–HCV RNA mixture. HCV RNA with no SSB proteins was used as the control.

Since SSB protein could bind ssRNA, determined if these four SSB proteins could protect RNA against nuclease attack. We first calculated the threshold dose of Benzonase against 120 ng of HCV RNA, and determined that 3.2×10^−3 ^U of Benzonase could partially digest 120 ng of HCV RNA at room temperature within 30 min, while 3.2×10^−2 ^U could completely digest 120 ng of HCV RNA (Figure 7). When SSB protein bound HCV RNA was digested with 3.2×10^−2 ^U of Benzonase at room temperature for 30 min, bl21- and kod-bound HCV RNA could withstand the digestion, ssob-bound HCV RNA was partially digested, and tth-bound HCV RNA was completely digested (Figure 7, middle left). When SSB protein-bound HCV RNA was digested with 3.2×10^−3 ^U of Benzonase at room temperature for 30 min, ssob-, bl21- and kod-bound HCV RNA could withstand the digestion, while tth was not protective against digestion (Figure 7, middle right, Table 1).

### SSB Proteins Affect Viral RNA Metabolism

**Figure 8:**
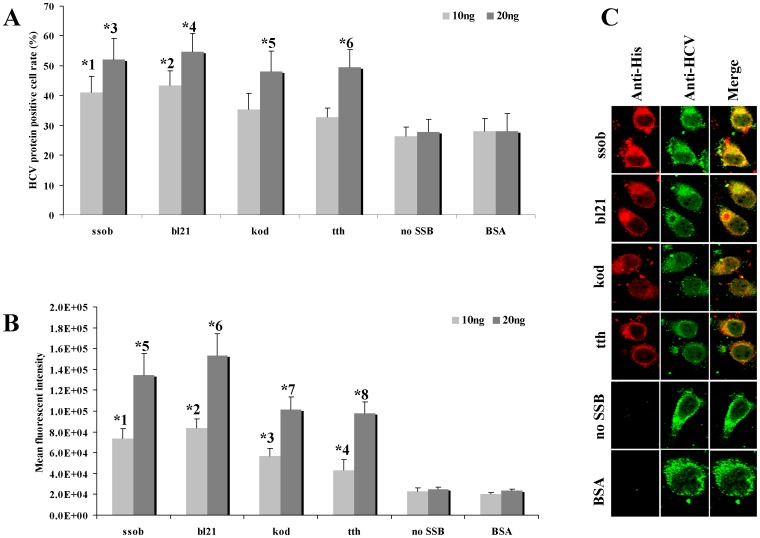
SSB proteins modulated HCV RNA metabolism. A. *In vitro*–transcribed HCV RNA (500 ng) was transfected into 1×10^6^ Huh7 cells with or without 10 or 20 ng of SSB proteins. The BSA was used as random protein control. After the 72-h incubation, the cells were harvested for RNA expression analysis. A HCV-infected patient serum was used as the primary antibody to probe HCV proteins expressed by transfected HCV RNA. The positive cells were visualised using a FITC-labelled goat anti-human antibody followed by FACS analysis. All *p* values marked by star are less than 0.05. B. The mean fluorescence intensity of the HCV protein positive cells were calculated by FACS. All *p* values marked by star are less than 0.05. C. cellular localization and co-localization analysis, the above cells were double stained with anti-HCV serum and anti-His (all SSB proteins were labelled with His-tag) antibody, localization of SSB proteins and co-localization of SSB and HCV protein were evaluated by a cofocal microscope.

Since SSB proteins could bind and protect viral RNA, we evaluated their influence on viral RNA metabolism. *In vitro* viral RNA expression was always reduced by RNase attack both during the transfection process and its life cycle after transduction into the host cell. Based on the molar quantities of both SSB proteins and viral RNA, we determined the optimal dose of viral RNA and SSB proteins. *In vitro*–transcribed HCV RNA (500 ng) was transfected into 1×10^6^ Huh7 cells with or without 10 or 20 ng SSB proteins, Bull serum albumin (BSA) was used as a random protein control. After a 72 h incubation, viral RNA transduction and expression was analysed using an immunocellularchemistry assay. A well-verified chronic HCV–infected patient serum was used as the primary antibody to probe HCV proteins expressed by transfected HCV RNA. After conjugation with FITC-labelled goat anti-human secondary antibody, the numbers of positive cells and mean fluorescence intensity were determined using FACS. As shown in Figure 8A, the HCV RNA with no SSB proteins or combined with BSA resulted in 27.8±4.3% and 28.0±4.2% HCV protein positive cells, while HCV RNA combined with 10 ng of either ssob, bl21, kod or tth resulted in 40.9±5.6%, 43.3±4.9%, 35.4±5.4%, and 32.8±3.0% HCV-protein-positive cells, respectively. When the SSB protein concentrations were increased to 20 ng, these rates increased to 52.1±7.1%, 54.6±6.2%, 48.1±6.7%, and 49.5±5.8%, respectively, while the HCV RNA with no SSB proteins or combined with BSA resulted in 26.4±3.1% and 28.0±5.8% HCV protein positive cells (Figure 8A). Taken together, these data suggested that SSB proteins could significantly enhance the viral RNA transduction efficiency (Table 1).

To evaluate whether the SSB proteins could enhance the viral protein expression, the mean fluorescence intensity of HCV protein positive cells were calculated. As shown in Figure 8B, the mean fluorescence intensity of the cells derived from HCV RNA with no SSB proteins or combined with BSA were 23234±3345 and 20425±1423, respectively. While the mean fluorescence intensity of the cells derived from HCV RNA combined with 10 ng of either ssob, bl21, kod or tth were 73434±9890, 83534±8971, 56564±7679, and 43434±9670, respectively. When the SSB protein concentrations were increased to 20 ng, these mean fluorescence intensities increased to 134344±21011, 153434±21098, 101234±12100, and 98000±10910, respectively, while the HCV RNA with no SSB proteins or combined with BSA, the mean fluorescence intensity were 24866±2443 and 23431±1412, respectively (Figure 8B). Taken together, these data suggested that SSB proteins could significantly enhance the viral protein expression (Table 1).

To confirm that SSB proteins were transfected into host cells along with the viral RNA, cells transfected with 500 ng HCV RNA and SSB proteins were analysed using the immunocellularchemistry assay 24 h after transfection. SSB proteins could be detected by the primary anti-His antibody followed by a Alexa Fluor 568-labelled secondary antibody in cells transfected with SSB proteins, which suggested that SSB proteins bound viral RNA and co-entered host cells with the transfection complex (Figure 8C).

## Discussion

The electrostatic, hydrogen-bonding and stacking between SSB proteins and nucleoside bases, sugars and phosphates of ssDNA was believed to form the basis for ssDNA binding and specificity [17]. In this study, our results showed that all four SSB proteins bind HCV RNA. When the SSB proteins were heated at 95°C for 9 h, ssob, kod and bl21 could still bind HCV RNA, and the SSB protein protected the bound RNA against Benzonase digestion. When these SSB proteins were included in the reverse transcription reaction mix, they increased first-strand cDNA synthesis yield more than five times (data not shown). Furthermore, our study demonstrated that SSB proteins improved the transduction and expression efficiency of *in vitro*–transcribed viral RNA. *In vitro*–transcribed HCV RNA acted as mRNA for viral protein translation in transfected host cells [18]–[20]. Our data suggested that the SSB proteins could not only bind the viral RNA and undergo co-entry into the host cell with the transfection complex, but also improve viral RNA function. These enhancements could be due to increased HCV RNA stability, increased translation efficiency, increased HCV RNA replication, and increased protein stability. Together with the enhancement of first strand cDNA synthesis, these effects should not only contribute to the RNA protection role, but may also be related to the biological functions of SSB proteins in RNA metabolism pathways in organisms [21]–[23]. Notably, the SSB proteins derived from bacteria displayed biological function in mammalian cells. Thus, this study has increased our knowledge of, and may reveal novel applications for, SSB proteins.

*Thermococcus kodakarensis* KOD1 is a novel thermophilic bacterium [24], the DNA polymerase derived from which exhibited high fidelity and long-term heat resistance [25]. To explore the possible application of its SSB proteins, we first expressed kod, and performed systemic functional studies together with the other three SSB proteins which were reported previously. Our results showed that kod binds ssDNA and viral RNA, and showed excellent heat resistance compared to the other SSB proteins. kod also significantly enhanced PCR specificity and improved RNA expression. Thus, kod may in future be used to enhance DNA amplification or protein expression.

The functional unit of bl21 was reported to be tetrameric [3], although the monomer migrated at approximately 20 kDa based on SDS-PAGE analysis, which is consistent with previous reports [3]. Our native PAGE analysis showed that bl21 always migrated at approximately 480 kDa, which is not consistent with other reports [2], [3], in these reports, the authors had not provided the data derived from native-PAGE, so it is possible that the native-PAGE condition could not damage the interaction between bl21 tetramers. For kod, there were always two bands migrating at approximately 40 kDa. Since no N-linked glycosylation site was identified in its primary sequence, this form might be the result of degradation. All SSB proteins expressed in this report were his-tagged at the N-terminus. Our data showed that the his-tag had no effect on the function of the SSB proteins, which is beneficial for industrial production.

The four SSB proteins were divided into two groups based on primary sequence analysis. tth and bl21 were similar to that of *E. coli* (NCBI LOCUS: 1EYG_D), which suggested that although TTH is a thermophilic bacterium, its SSB shares structural features with that of *E. coli*. kod is similar to *Sulfolobus solfataricus*, which suggested that these proteins from both thermophilic bacteria are relatively similar. Protein derived from non-extremophiles showed moderate heat-resistance. Interestingly, protein derived from thermophilic bacteria was not always heat-resistant.

Although ssRNA could be stained with ethidium bromide in a TAE gel, visualisation of the ssDNA protein complex was difficult. Ethidium bromide could not efficiently stain the ssDNA, but ssDNA was stained red by AgNO_3_. Thus, we performed a series of experiments to explore the characteristics of ssDNA and ssDNA-binding patterns by AgNO_3_ staining. In this report, we used Coomassie brilliant blue-stained gels to represent a series of experiments since the binding activity could be reflected by mobility changes of the protein ssDNA complex. Regarding the effects on PCR specificity and enhancement of reverse transcriptions, SSB proteins were mixed with DNA polymerases and reverse transcriptases *in vitro*. We found that the SSB proteins did not interact with the DNA polymerase or reverse transcriptase (data not shown). Thus, the effects of SSB proteins on PCR specificity and enhancement of reverse transcriptions is likely accomplished through other mechanisms, and not by a direct interaction with the DNA polymerase and reverse transcriptase.

*E. coli* SSB was found to increase the processivity of DNA unwinding by the HCV helicase, SSB stabilizes the helicase at the unwinding junction and prevents its dissociation [26]. Together with our data, host SSB proteins might be involved into viral life cycle and viral pathogenesis.

In summary, we present here a systematic functional analysis of a novel SSB protein derived from KOD1 and three reported SSB proteins. We found that all SSB proteins bound to ssDNA and viral RNA. Also, ssob and bl21 resisted heat-treatment for more than 4 h, and kod could withstand 95°C for at least 10 h. When SSB proteins bound to RNA, they both protected the RNA against nuclease attack and accelerated viral RNA processes in the host cell. Our results may provide a basis for future studies of the novel functions of SSB proteins.
