# Correction: Systematic Functional Comparative Analysis of Four Single-Stranded DNA-Binding Proteins and Their Affection on Viral RNA Metabolism

**DOI:** 10.1371/annotation/b45ca472-8c85-407c-90e4-59f0aff300ea

**Published:** 2013-08-08

**Authors:** Haiyan Shi, Yonghui Zhang, Guohui Zhang, Jinlei Guo, Xun Zhang, Haiyan Song, Jianxin Lv, Jimin Gao, Yuepeng Wang, Litian Chen, Yue Wang

A second grant from the Natural Science Foundation of Zhejiang Province and a grant from the National Natural Science Foundation of China were incorrectly omitted from the Funding Statement. The first sentence of the Funding Statement should read: This work is partially supported by grants from the National Natural Science Foundation of China (81170302), and by grants from the Natural Science Foundation of Zhejiang Province (Y2101159 and Y2101019). 

